# Measles 2018: a tale of two anniversaries

**DOI:** 10.15252/emmm.201809176

**Published:** 2018-04-23

**Authors:** Philippe J Sansonetti

**Affiliations:** ^1^ Unité de Pathogénie Microbienne Moléculaire & Unité INSERM 1202 Institut Pasteur Paris Cedex 15 France; ^2^ Microbiologie et Maladies Infectieuses Collège de France Paris France

**Keywords:** Immunology, Microbiology, Virology & Host Pathogen Interaction

## Abstract

This year is the 50^th^ anniversary of the reduction in measles in the USA, following introduction of general vaccination, but also the 20^th^ anniversary of a now retracted research paper that suggested a link between the measles–mumps–rubella (MMR) vaccination and autism, which contributed to falling vaccination rates and re‐emergence of measles cases globally.

Hurl your calumnies boldly, something is sure to stick ‐Francis Bacon

For measles, 2018 is the year of two anniversaries that illustrate a medical paradox:

The happy 50^th^ anniversary of the collapsing incidence of measles in the USA, following 5 years of a nationwide implementation of the new vaccine; and the sad 20^th^ anniversary of a research paper claiming a link between the measles–mumps–rubella (MMR) vaccination and an occurrence of autism in 12 children (Wakefield *et al*, 1998), a publication that is likely to be linked to undermined public confidence in the measles vaccine and in vaccinations in general.

Let's consider the elements of this paradox that threaten the efforts, hope, and—until recently—prediction that measles was on track to be the third eradicated communicable disease after smallpox and poliomyelitis.

In 1968, 5 years after licensing and large implementation of the first measles vaccine in the USA, the number of annual cases of the disease briskly collapsed to a historical number of 22,231, compared to about 500,000 annual cases (under‐evaluated by insufficient reporting) that caused 400–500 deaths annually in the preceding decade (Morbidity and Mortality Weekly Report, 1971, https://www.jstor.org/stable/i40169561). During the pre‐vaccination period, the disease was so prevalent that people came to accept the false notion that “measles is an obligatory but benign disease, better catch it to be protected”, the *vox populi* cliché. For good reason, this was not written in textbooks about paediatric infectious diseases; beyond a relatively low mortality rate in high‐income regions compared to other epidemics (*ca*. 1/1,000 cases) due to antibiotics that efficiently cured potentially lethal bacterial pulmonary superinfections, global mortality rates, including low‐income regions, reached 2.6 million annually during this period, making measles the first cause of paediatric mortality in these areas (http://www.who.int/mediacentre/factsheets/fs286/en/). In addition, the disease burden was—and still is—marked by severe morbidity, not only bacterial superinfections facilitated by the immunosuppressive potential of the measles virus, but also complications due to the virus itself: acute laryngitis, pneumonia, severe diarrhoea and neurological complications, particularly blindness, deafness, acute encephalitis and the disastrous late‐occurring subacute sclerosing panencephalitis (SSPE). Recent studies in California showed that SSPE is in fact even more common than previously assumed (see Box A).

**Box A: Further reading**

Antona D, Lévy‐Bruhl D, Baudon C, Freymuth F, Lamy M, Maine C, Floret D, Parent du Chatelet I (2013) Measles elimination efforts and 2008–2011 outbreak, France. *Emerg Infect Dis* 19: 357–364

Black C, Kaye JA, Jick H (2002) Relation of childhood gastrointestinal disorders to autism: nested case‐control study using data from the UK General Practice Research Database. *BMJ* 325: 419–421

Fredricks DA, Relman DA (1996) Sequence‐based identification of microbial pathogens: a reconsideration of Koch's postulates. *Clin Microbiol Rev* 9: 18–33

GBD 2016 Causes of Death Collaborators (2017) Global, regional and national age‐sex specific mortality for 264 causes of death, 1980–2016: a systematic analysis for the Global Burden of Disease 2016. *Lancet* 390: 1151–1210

Hotez PJ (2018) «Melanie's measles» is deadly and causes permanent neurologic impairment. *Microbes Infect* 20: 63–64

Larson HJ, de Figueiredo A, Xiahong ZX, Schulz WS, Verger P, Johnston IG, Cook AR, Jones NR (2016) The state of vaccine confidence 2016: global insight through a 67‐country survey. *EBioMedicine* 12: 295–301

Leuridan E, Sabbe M, Van Damme P (2012) Measles outbreak in Europe: susceptibility of infants too young to be immunized. *Vaccine* 30: 5905–5913

Madsen KM, Hviid A, Vestergaard M, Schendel D, Wohlfahrt J, Thorsen P, Olsen J. (2002) A population‐based study of measles, mumps and rubella vaccination and autism*. N Engl J Med* 747: 1477–1482

Weber T, Frye S, Bodemer M, Otto M, Lüke W (1996) Clinical implications of nucleic acid amplification methods for the diagnosis of viral infections of the nervous system. *J Neurovirol* 2: 175–190

Wendorf KA, Winter K, Zipprich J, Schechter R, Hacker JK, Preas C, Cherry JD, Glaser C, Harriman K (2017) Subacute Sclerosing Panencephalitis: the devastating measles complication that might be more common than previously estimated. *Clin Infect Dis* 65: 226–232

Wilson K, Mills E, McGowan J, Jadad A. (2003) Association of autistic spectrum disorder and the measles, mumps and rubella vaccine. *Arch Pediatr Adolesc Med* 157: 628–634

No! Measles was not and will never be a benign disease that any child should have to experience (see Box A). It is a major paediatric threat that fully justified the intensive efforts to develop a vaccine—full stop. “Melanie's Marvelous Measles”, a 2011 self‐published children's book claiming that measles is beneficial to health, is a sad joke that puts children in danger by misleading unsuspecting parents.

Sustained efforts in the following three decades have allowed US health authorities to consider measles eliminated from their country in 2000. Elimination was obtained at the cost of achieving large vaccine coverage and introducing a second dose of the vaccine, a conjunction deemed to guarantee elimination (Rosenthal & Clements, 1994). Measles is an ultrasensitive and merciless marker of insufficient vaccine coverage. Indeed, the measles virus high capacity of inter‐individual transmission (i.e. R0 = 15–20) demands high vaccine coverage (i.e. 1 – 1/R0 = 95% for the first dose). Similar results were eventually obtained in Europe by developing seemingly ambitious vaccination policies aiming at disease elimination. Elimination rather than eradication is all that can be achieved as long as the virus is maintained in pockets of unvaccinated populations, or in a context of globally insufficient vaccine coverage, reintroduced from the outside, thus allowing residual virus circulation. Comparative dynamics of measles in non‐immunized and partially immunized populations have been the subject of intensive mathematical modelling that helped rationalize public health interventions (Jansen & Stollenwerk, 2005).

In spite of progress, measles remained at the front line of paediatric mortality and morbidity in low‐income countries in 2000, still accounting for a million deaths per year; subsequent intensive efforts, particularly by GAVI under the auspices of WHO, brought this number down to an historically low estimate of 68,000 in 2016, an 84% decrease (see Box A), hence retrograding measles from the top three to the 13^th^ rank among children killers on a worldwide basis (see Box A), and demonstrating that elimination of measles was also possible in the most impoverished populations of the planet. Hence, we can in principle still contemplate the tantalizing goal of making measles the third eliminated and possibly eradicated communicable disease after smallpox and polio.

Yes, we might have still been able to hope for that goal, if it were not for the sad anniversary in 2018: 20 years ago Andrew Wakefield, a gastroenterologist with indisputable expertise in inflammatory bowel diseases (IBDs) at the Royal Free Hospital in London, published in the *Lancet* journal a highly unexpected syndrome, associating ileal lymphoid hyperplasia, non‐specific colitis and pervasive developmental disorder occurring in previously healthy children (Wakefield *et al*, 1998). This publication followed a previously published cohort study indicating that measles vaccination was a risk factor for IBDs (i.e. Crohn's disease and ulcerative colitis; Thompson *et al*, 1995). This new entity, according to Wakefield, “was generally associated in time with possible environmental triggers”. The environmental trigger, according to the parents’ anamnesis, was the recent administration of MMR vaccine. Indeed, as might be expected, the measles component became the primary suspect despite poor exploration of alternative infectious aetiologies, particularly enteroviruses, as some of them were known for their neurological tropism and for which molecular diagnostic had been validated (see Box A), and a lack of demonstration of the presence of the measles vaccine virus in the pathological samples (i.e. intestinal biopsies, hyperplastic lymph nodes and cerebro‐spinal fluid). Even without the knowledge of the author's undeclared conflict of interests with anti‐vaccine lobbyists at the time of submission, it remains a mystery how this manuscript could have been published, given its likely impact on public health, without minimally demanding the slightest shred of evidence that the measles vaccine strain fulfilled—even partially—Koch's criteria of causality by finding pathogen nucleic acid sequences in pathological samples. Neither the authors nor the reviewers of this manuscript could plausibly have been ignorant of the published article by Fredricks and Relman that revisits Koch's postulate of causality in the light of sequence‐based identification of microbial pathogens published just 2 years earlier, which was at the time a crucial analysis to alleviate the controversy raised regarding HIV being the aetiological agent of AIDS, according to the notion of “scientific concordance” (see Box A). This is even more surprising, as Wakefield and his collaborators had concurrently developed a sensitive method to detect measles virus genomic RNA by combining hybrid capture, reverse transcription and PCR which, by the way, did not identify measles signatures in IBD (Chadwick *et al*, 1998). In spite of the fundamental lack of molecular information, the discussion essentially focussed on the hypothesis of the vaccine's responsibility for the symptoms. The authors even stated in the discussion: “we did not prove an association between measles, mumps, and rubella vaccine and the syndrome described. Virological studies are underway that may help to resolve this issue”. These were never published. Had they been positive—unlike those in IBD—it is hard to believe the authors would have hesitated to communicate them with due urgency!

The article immediately caused an avalanche of reactions among experts, mostly negative, particularly in the form of letters to the *Lancet* on the central issue: “time‐correlation does not imply causality; B following A does not mean that A caused B…”. Others, however, claimed that this study had opened the way to something we would today call the gut–brain axis or that they had themselves detected measles virus in pathological products in similar situations, but these claims invariably related to individual, uncontrolled cases.

All ingredients of the emerging post‐truth ideology were crystallized in this textbook example of overstated claims and ill‐supported findings, with autistic children and their parents as defenceless victims, and the vaccine industry and their academic lackeys portrayed as profiteering villains. Welcome to the world of fake news in which the authority of scientific evidence loses ground to alternative truths and science.

None of the subsequent studies conducted with irreproachable epidemiological methodology (Donald & Muhtu, 2002), including a nationwide paediatric cohort in Denmark over a 10‐year period (see Box A) and a meta‐analysis of properly controlled studies (see Box A) were able to detect a correlation between an increased occurrence of autism and the MMR vaccine. Nor could they demonstrate a link between autism and inflammatory gastrointestinal diseases (see Box A). More recent studies benefiting from longer period of evaluation confirmed the lack of association (Stratton *et al*, 2012; Modabbernia *et al*, 2017).

Again: why was this paper ever published? Why did the system of academic self‐control and that of journal publishing fail? The study showed enough weaknesses regarding its epidemiological insignificance with such a limited sample (*n* = 12) of selected individuals, uncontrolled design and its unconvincing physiopathological dimension. Why did it eventually take a talented investigative journalist, Brian Deer of the Sunday Times, to reveal the massive conflict of interests of Wakefield and some of his collaborators, and to disentangle some fraudulent treatment of samples and data to prepare the ground for retraction? Why did it take 12 years for the *Lancet* to publish this long‐overdue retraction—6 years after Deer's first report? (Wakefield *et al*, 2010; Deer B. Revealed: MMR research scandal. *Sunday Times* 2004 Feb 22). Too late: the worm was in the fruit. The Wakefield *et al* paper ignited a heated debate in spite of overwhelming scientific evidence against a causative link between measles vaccination and autism. It started in the UK and quickly spread to continental Europe, North America and Australia, and more recently Asian countries such as Japan. Which parents possessing average scientific knowledge would not be scared to immunize their beloved child with a product that might cause autism? Unfortunately, the doubt was viciously introduced in these parent's minds and would remain imprinted, even strengthened by indiscriminate exposure to fake news in the unchecked space of social media. Indeed, the growing vaccine hesitancy did not remain restricted to measles or MMR vaccines, already encompassing all vaccines, questioning both their safety and efficacy (see Box A). By now, Andrew Wakefield has become the iconic hero of anti‐vaccination militants.

Not unexpectedly, it did not take long for measles epidemics to reappear and increase in frequency and size (Ramsay, 2003) as a logical consequence of insufficient vaccine coverage in agreement with modelled scenarios. Europe has clearly entered an era of measles re‐emergence. France experienced 24,000 cases between 2008 and 2012, with about 1,000 hospitalizations in ICUs for severe lower respiratory tract infections, 35 cases of encephalitis and 15 deaths (see Box A). In 2011, at the peak of the epidemic, France accounted for half of the cases in Europe. A majority of the cases corresponded to not yet vaccinated infants (see Box A). Others were unvaccinated or insufficiently vaccinated individuals (one dose only), including adults. This emphasized the herd effect of high vaccine coverage offering protection to the non‐vaccinated—often fragile—individuals. Between 1 February 2017 and 31 January 2018, 14,732 cases of measles were reported to the European Surveillance System (ECDC) by 30 EU/EEA countries. Most cases were reported by Romania (5,224), Italy (4,978), Greece (1,398) and Germany (906) (ECDC Report, 2018, https://ecdc.europa.eu/en/publications-data?%20f%5B0%5D=publication_series%3A2702). In spite of national actions to catch up, according to data collected by WHO, vaccination coverage remains too low in several EU/EEA countries to reach elimination (http://www.who.int/immunization/monitoring_surveillance/data/en/).

Here is the paradox. Do we need to rediscover the disastrous impact of preventable infectious diseases on children to rediscover the value of vaccines?

## Conflict of interest

The author declares that he has no conflict of interest.
